# Neuropeptides in asthma, chronic obstructive pulmonary disease and cystic fibrosis

**DOI:** 10.1186/s12931-018-0846-4

**Published:** 2018-08-06

**Authors:** Kalina R. Atanasova, Leah R. Reznikov

**Affiliations:** Department of Physiological Sciences, College of Veterinary Medicine, University of Florida, 1333 Center Drive, PO Box 100144, Gainesville, FL 32610 USA

**Keywords:** Neuropeptides, Lung diseases, Mucus, Cystic fibrosis, Asthma, COPD

## Abstract

The nervous system mediates key airway protective behaviors, including cough, mucus secretion, and airway smooth muscle contraction. Thus, its involvement and potential involvement in several airway diseases has become increasingly recognized. In the current review, we focus on the contribution of select neuropeptides in three distinct airway diseases: asthma, chronic obstructive pulmonary disease (COPD), and cystic fibrosis. We present data on some well-studied neuropeptides, as well as call attention to a few that have not received much consideration. Because mucus hypersecretion and mucus obstruction are common features of many airway diseases, we place special emphasis on the contribution of neuropeptides to mucus secretion. Finally, we highlight evidence implicating involvement of neuropeptides in mucus phenotypes in asthma, COPD and cystic fibrosis, as well as bring to light knowledge that is still lacking in the field.

## Background

Lung diseases are among the top 5 mortality-causing diseases in the World [1]. As with many organs, lung diseases vary significantly in their etiology and pathology, ranging from inherited genetic diseases like cystic fibrosis (CF), to allergic and inflammatory disorder diseases, such as asthma and chronic obstructive pulmonary disease (COPD). Although hallmark features of asthma, COPD, and CF differ, mucus obstruction is a common attribute among all [2]. Studies examining asthma, COPD and CF have focused on the role of the immune system [3–7] and epithelial ion channels in mucus secretion [8–12], but the involvement of the nervous system, and in particular neuropeptide signaling, remains a field of much unknowns [13]. This is important given the rich innervation of the airway and its modulatory role in mucus secretion [14–19]. The goal of this review is to summarize the current knowledge on the effects of select neuropeptides in the lungs, with an emphasis on asthma, COPD and CF. A specific interest is placed on neuropeptide-mediated regulation of mucus secretion and their effects on the expression of the two major secreted gel-forming mucins in the airway, mucin5AC (muc5AC) and mucin5B (muc5B) [20].

### Neuropeptides production and secretion

Neuropeptides, by definition, are peptides that are formed by the enzymatic processing of gene-encoded precursor molecules [21]. They are produced, stored, and secreted upon demand via regulated secretory pathways. Due to different enzyme cleaving and processing of the precursor molecules, current neuropeptides are classified into families based upon the genes encoding those precursors [21, 22]. The molecules that fit this definition are called “classic neuropeptides”. However, with increased interest and evolving research, new members are constantly emerging, and the definition continues to expand as new molecules that have some neuropeptide features, but lack others, are discovered [21]. Expression of precursor molecules occurs predominantly in neurons where they are stored in large granular vesicles in the cytoplasm and released upon stimulation [21, 23]. After their release, classical neuropeptides exert their specific actions upon a variety of target cells via G-protein coupled receptors [21, 23]. Their actions can be exerted on other neurons as modulators of signaling, or on non-neuronal cells as signaling molecules. Therefore in many organs, neuropeptides can exert effects through direct innervation of the end organ (synaptic contact), but also through non-synaptic contact and paracrine activity on neighboring cells [21]. Additionally, more than one receptor type and different G-protein coupling of neuropeptide receptors in different tissues lead to variable effects of the same neuropeptides in different tissues/cell types [24–36]. This also adds to the complexity observed in the effects of neuropeptides.

Many neuropeptides co-exist in the same neurons, where they influence production and secretion of one another, thus exerting a neuromodulatory role [21, 23, 37]. Because of this, predicting the consequences of neuropeptide release and/or activation can be difficult. Moreover, in the lung, non-neuronal cells, known as neuroendocrine cells, synthesize and secrete neuropeptides. These cells add an additional layer of regulation and have recently gained interest in asthma and CF [38–44].

### Neuropeptides in asthma, COPD and cystic fibrosis

Asthma and COPD are common, chronic, and heterogeneous pulmonary diseases that have a significant impact on quality of life [45]. Asthma is primarily viewed as an inflammatory disorder of the airways and often is diagnosed at young age [37]. It is characterized by wheezing, cough, chest tightness and variable airflow limitation that is partially reversible [37, 46]. Key features of asthma include airway hyperreactivity, as well as alterations in the quantity and quality of airway mucus [18, 47, 48]. Although inflammation is a cornerstone of asthma [49], several studies have shown that the nervous system plays a fundamental role in its pathogenesis [50–53].

Unlike asthma, COPD almost exclusively affects adult populations and is often related to long-term exposure to tobacco smoke or other chemicals [54–56]. Inflammation, persistent airflow limitation, and mucus obstruction are also salient features [57]. The role of the nervous system in COPD is still being elucidated [58–61].

In contrast to both asthma and COPD [62], CF is a single gene disorder that arises from mutations in the *cystic fibrosis conductance regulator (CFTR)* gene [63]. Mutations in CFTR result in faulty ion transport, which in the airway impairs several key airway host defenses (reviewed here [64, 65]. Features of CF include mucus obstruction and recurrent airway infections. Neural involvement in CF has been proposed [66–68].

**Fig. 1:**
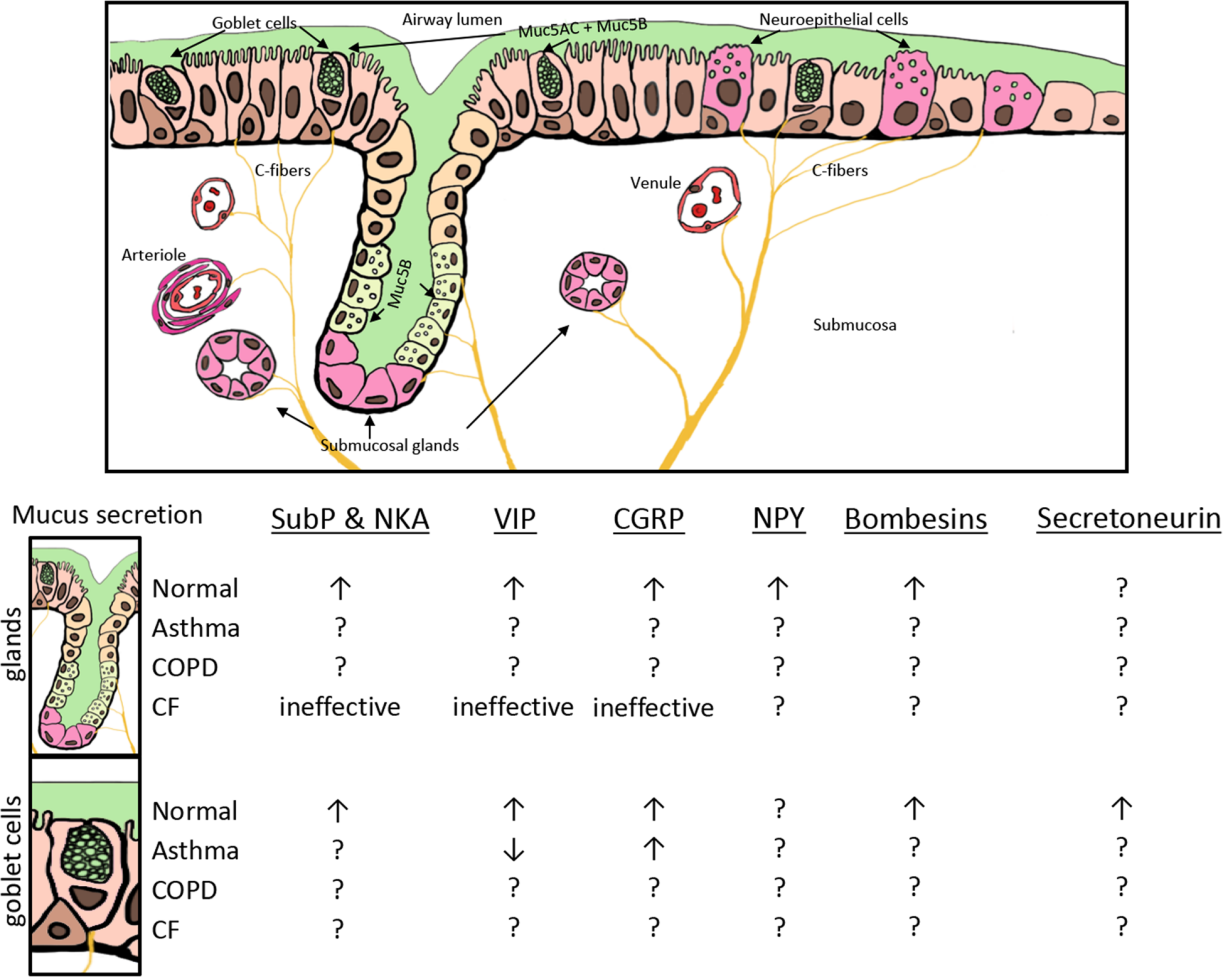
Summary of the effects of neuropeptides on mucus secretion from submucosal glands and goblet cells. Diagram shows model of airway. An up arrow indicates increased secretion, a down arrow indicates decreased secretion. A question mark indicates effect unknown. Abbreviations: SubP, substance P; NKA, neurokinin A; VIP, vasoactive intestinal peptide; CGRP, calcitonin gene-related peptide; NPY, neuropeptide Y; COPD, chronic obstructive pulmonary disease; CF, cystic fibrosis

**Fig. 2:**
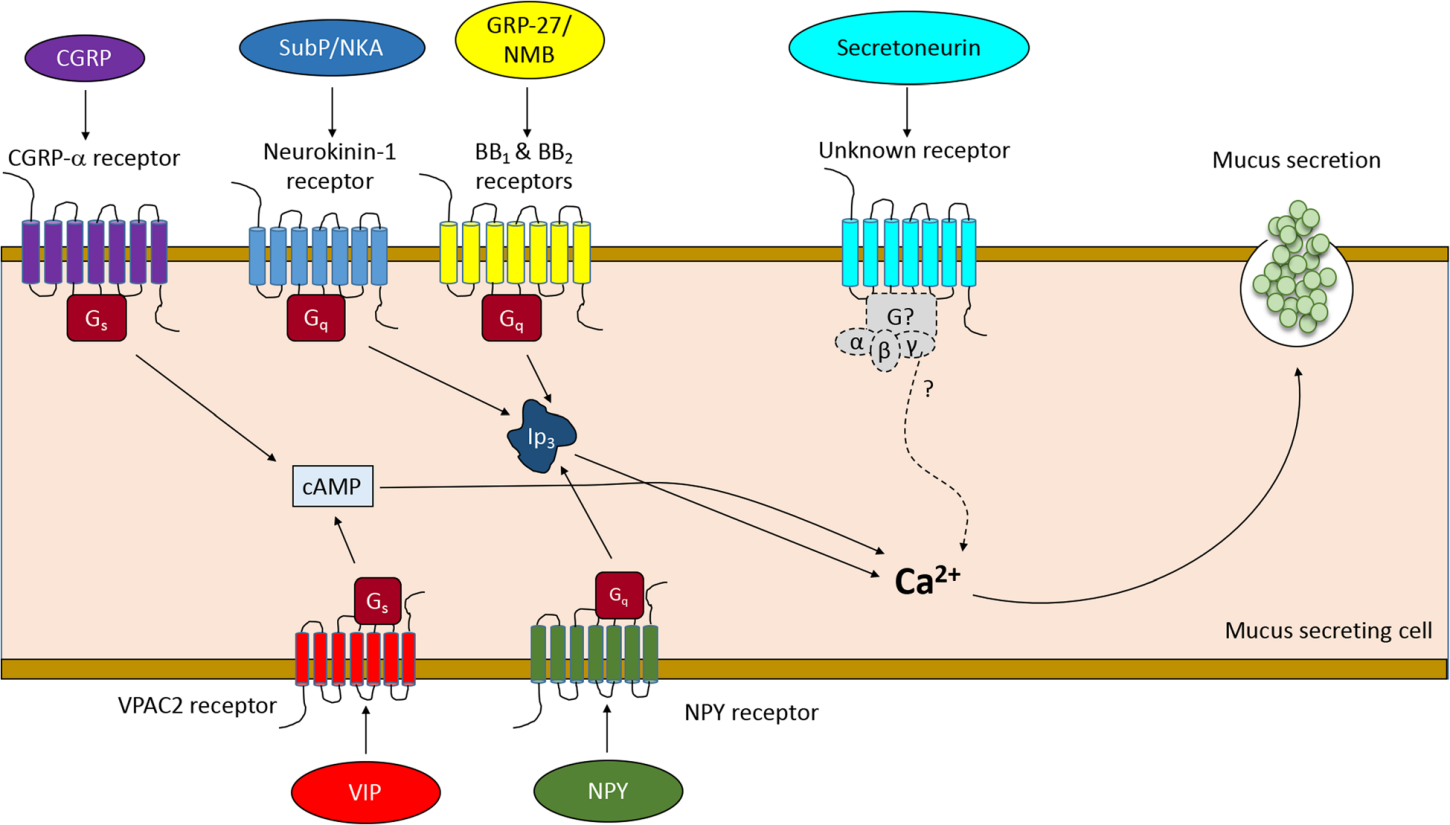
Simplified schematic of select neuropeptide and their proposed receptor mechanisms mediating mucus secretion. Receptors coupled to G_s_ increase cAMP through adenylyl cyclase (not shown). cAMP then increases intracellular Ca^2+^ through downstream mediators, such as protein kinase A (not shown). Receptors coupled to G_q_ lead to breakdown to inositol 1,4,5-phosphate (IP3) and subsequent mobilization of Ca^2+^ from intracellular stores. Ca^2+^ serves as a common mediator of mucus granule discharge and exocytosis [231]. Additional details regarding receptor and effector mechanisms are shown in Table 3. Abbreviations: SubP, substance P; NKA, neurokinin A; VIP, vasoactive intestinal peptide; CGRP, calcitonin gene-related peptide; NPY, neuropeptide Y; GRP, gastrin-releasing peptide; NMB, neuromedin B; IP3, inositol 1,4,5-trisphosphate; VPAC2, vasoactive intestinal peptide receptor 2; bombesin subtype-1 receptor (BB_1_); bombesin subtype-2 receptor (BB_2_)

Here we review a few select neuropeptides that have either been shown to impact or have the potential to impact the pathogenesis and progression of either asthma, COPD, or CF. Special emphasis is placed on neuropeptides that have not received much attention, and/or those that have been recently discovered. We summarize the involvement of these neuropeptides in mucus secretion in Table 1, and their effects on mucus secretion in asthma, COPD, and CF in Fig. 1. Further, a summary of the expression and/or release of these neuropeptides in people with asthma, COPD, or CF is provided in Table 2. Fig. 2 and Table 3 provide a summary of the known/proposed G-protein coupled receptors mediating the effects of the select neuropeptides.

**Table 1:** General overview of neuropeptides and their effect on airway mucus secretion

Neuropeptide	General effect on mucus secretion
Calcitonin gene-related peptide (CGRP)	Induced small concentration-dependent increases in basal mucus volume, lysozyme and albumin outputs from in vitro ferret trachea culture at baseline [143].
	Stimulated goblet cell hyperplasia when co-administered with GABA [39].
	Stimulate goblet cell secretion [142]
Bombesins	GRP-27 induced dose-dependent increase of respiratory glycoconjugate secretion in feline tracheal organ culture [201].
	Bombesin receptor-activated protein BRAP (a downstream protein from the activation of the orphan bombesin receptor subtype-3) regulates neutrophil elastase-induced muc5AC hypersecretion in human bronchial epithelial cell line [207].
Substance P (SubP)	Stimulates human airway submucosal gland secretion [76, 79].
	Increases goblet cell secretion [142].
Granins	Secretoneurin induced Muc5AC hypersecretion in a dose- and time-dependent manner in human HBE16 bronchial epithelial cell line [36].
Vasoactive intestinal peptide (VIP)	Stimulates mucus secretion in ferret trachea [113].
	Knockout of the VIP receptor (VPAC2) in a murine model of Aspergillus antigen-induced asthma lead to a marked enhancement of MUC5AC mRNA and an associated increase in goblet cells in the lungs [232].
Neuropeptide Y	Modulates mucus output from airway submucosal glands [173, 174].

**Table 2:** ᅟ

		SubP	NKA	VIP	CGRP	NPY	Bombesins	Granins
Asthma	Expression or concentration	↑	↑	↓	↑	↑ ↓	↑	↑
	Details	↑ levels in sputum [81, 82] & BAL [83, 233], ↑ innervation, [149, 234] ↑ neurons [235]	Sputum muc5AC and NKA positively correlate [84], ↑ neurons [235]	↓ innervation [116, 149]	↑ airway expression [148, 150]; ↑ innervation [149]; ↑ sputum[157]	↑ in BAL [170], ↑ plasma/serum [175, 177, 219]; ↑ innervation [236] ↓ smooth muscle [180]	↑ in airway[39] ↑ expression in lungs in risk factors for asthma [204, 205]	↑ in serum, mixed population, including some asthmatics [218]
COPD	Expression or concentration	↑ ↓	↓ ↑	↑	↑	↓ ↑	↑	↑
	Details	↑ levels in sputum [89]; ↓ sputum concentrations during exacerbation [90]	↓ sputum concentrations during exacerbation [90]; ↑ release in airways *ex vivo* [237]	↑ innervation in chronic bronchitis [127]; ↑ serum [124]	↑ sputum [157]; ↓ receptor expression in airways [158];	↓ smooth muscle [171]; ↑ innervation in risk factor for COPD [185];	↑ release from neuroepithelial cells [193]	↑ in serum [224]
CF	Expression or concentration	unknown	unknown	↓	↑	↑	↑	unknown
	Details	normal serum range: <20 -125 pg/ml (mean: 41 ±7 ), CF range: 21-170 pg/ml, (mean: 78 ± 15.8) [238]	unknown	↓ epithelium and alveolar wall [131]	↑ submucosal glands [161]	↑ in olfactory epithelium [191]	↑ in neuroepithelial cells [214]	unknown

**Table 3:** Receptor mechanisms of select neuropeptides

Neuropeptide	Receptor	Effectors & signaling molecules involved
Substance P/ Neurokinin A	neurokinin-1,2 & 3 receptors (NK-1,2 &3) (discussed and reviewed in [239] and [240]).	G_s_ and G_q_ [241-243]. ↑ intracellular Ca^2+^, DAG, cAMP, IP3 [79, 239, 244-246]. CFTR has been implicated [100].
VIP	VPAC1, VPAC2 and PAC1 [30, 247].	G_s_, G_i_ and G_q_ [248, 249]. ↑ intracellular Ca^2+^, DAG, cAMP, IP3, [249]. CFTR has been implicated [132].
CGRP	CGRP-α subtype and CGRP-β subtype [31, 138, 250].	G_q_ and G_s_ [251]. ↑ intracellular Ca^2+^, DAG, IP3, cAMP [251, 252].
NPY	Y-1, Y-2, Y-4 and Y-5 receptors [32, 34, 182].	G_i_ and G_q_ [182, 253]. ↑ intracellular Ca^2+^, DAG, cAMP, IP3, [182, 253-256].
Bombesins (GRP-27; NMB)	NMB = subtype 1 receptor (BB_1_); GRP-27 = subtype 2 receptor (BB_2_); subtype-3 (BB_3_) = orphan [35].	G_i_ and G_q_ [35]. ↑ intracellular Ca^2+^, DAG, cAMP, IP3 [35, 257].
Chromogranins (Secretoneurin)	Currently suspected, but not identified G-protein coupled receptor.	↑ intracellular Ca^2+^, cAMP [36].

### Tachykinins

Tachykinins are a group of neuropeptides that are enzymatically cleaved from precursor proteins to form 10-12aa biologically active products [69]. The two major gene products that encode tachykinins are the preprotachykinin A (*TAC1)* gene – the parent of Substance P (SubP) and neurokinin A (NKA), and preprotachykinin B (*TAC3*) gene – encoding the precursor of neurokinin B (NKB) [21, 69, 70]. Of these, Sub P is the best studied. SubP and NKA, but not NKB, are localized to sensory nerves that are situated beneath and within the airway epithelium (C-fibers), around blood vessels, and to a lesser degree within airway smooth muscle [37, 71]. Their effects are mediated via NK1 receptors (mainly for SubP) and NK2 receptors (activated by NKA). Activation of these receptors induces airway smooth muscle constriction in humans, although the effects of SubP are variable [72–75]. SubP also has a direct effect on airway goblet cells and submucosal glands through NK1 receptor, causing mucus secretion [76–79]. Using receptor antagonists against NK1 and NK3 receptors in porcine tracheal explants, Philips and colleagues showed that SubP and NKB induced submucosal gland fluid flux, while NKA had no effect on the gland flux [80]. Their results also suggested that NK1 and NK3 receptors may induce glandular effects by different mechanisms – NK3 receptors are likely inducing activation of parasympathetic nerves, while NK1 may have direct effect on the glands [80].

#### Asthma

In asthmatic airways, mucus content positively correlated with SubP expression; NK1 receptors were also elevated with strongest expression detected on goblet cells [81]. The authors of that study concluded that neurogenic mechanisms contributed to asthma. Consistent with that, Tomaki and colleagues found that SubP levels in sputum correlated with airway obstruction in asthma [82]. In an experimental murine model of allergic asthma, increased bronchoalveolar lavage fluid concentrations of SubP were associated with induction of muc5AC mRNA [83], further suggesting a potential pathogenic role for tachykinins in asthma. Other studies have found a positive correlation between muc5AC and NKA protein expression in the sputum of asthmatics [84]. There are currently no studies available that provide information regarding the role of NKB in regulating muc5AC or muc5B expression, nor of SubP or NKA in the regulation of muc5B, in asthma. However, given that muc5AC is increased and muc5B decreased in the sputum obtained from asthmatics [48], it is possible (although speculative) that tachykinins have stimulatory roles on muc5AC, but inhibitory roles on muc5B.

Therapeutically, tachykinin receptor antagonists have been explored in asthma, with some reports suggesting beneficial effects (reviewed here [85]). For such studies, bronchoconstriction, inflammation and lung function were primary endpoints. For example, Van Schoor and colleagues found that SR 48969, a NK2 receptor antagonist, prevented bronchoconstriction provoked by NKA in mild asthmatics [86]. However, more recently, the NK1/NK2 receptor antagonist AVE5883 has provided mixed results, in which it mitigated NKA-mediated bronchoconstriction, but augmented allergen-induced decreases in forced expiratory volume (FEV_1_) in people with asthma [87]. Because decreases in FEV_1_ could be due to either bronchoconstriction and/or mucus obstruction, it is possible that dual blockade of NK1 and NK2 receptors in asthmatics augmented mucus secretion and/or bronchoconstriction to allergen. Therefore, examining expression of muc5AC and muc5B in the sputum of people with asthma provided tachykinin receptor antagonists might be especially important. Finally, despite some evidence that tachykinin receptor antagonists might be beneficial, they have failed to reach the market for asthma [88].

#### COPD

One report suggests that the concentration of SubP is elevated in the sputum of COPD patients [89], and it was suggested that neurogenic inflammation might contribute to the airway narrowing in COPD. Interestingly, however, in individuals with COPD, sputum SubP and NKA decreased during exacerbation [90]. The authors proposed that continual stimulation of sensory nerves during an exacerbation might have led to neuropeptide depletion, thus accounting for the decreased content. Additional studies have shown that cigarette smoke induces mucus secretion of goblet cells through activation of sensory nerve fibers [91], with SubP being the proposed mediator. Thus, since smoking is a major risk factor for COPD [92, 93], these findings suggested that tachykinin antagonists might be beneficial for COPD [29]. Furthermore, if SubP does mediate cigarette-induced secretion of goblet cells, then it is possible that it might also contribute to increased expulsion of mucus in COPD airways and/or regulate muc5AC and muc5B expression.

Therapeutically, tachykinin receptor antagonists have been shown to decrease inflammation in animal models of COPD [94]. Specifically, the number of macrophages and dendritic cells was decreased in the lung lavage fluid of mice exposed to cigarette smoke and provided the tachykinin receptor antagonist AVE5883. It is also interesting to note that several studies have found a pro-relaxing effect of SubP and NKA human blood vessels [95]. From this perspective, tachykinin antagonists might be of detriment in subpopulations of people with COPD (e.g., those that have pulmonary hypertension). This might explain why none of the tachykinin receptor antagonists that were in development for COPD [96] have exhibited a clear therapeutic benefit.

#### Cystic fibrosis

Although SubP stimulates gland secretion in “normal” submucosal glands, reports suggest that it is ineffective in people with CF [76]. This finding has been reproduced in pigs with CF [97]. An implication from those studies was that defective responses to SubP might contribute to airway pathology in CF. Given that glandular secretion in response to SubP is defective in CF, a speculation is that SubP-mediated secretion in CF might be associated with enhanced muc5AC to muc5B secretion ratios, effectively mimicking asthma [98]. Moreover, in contrast to asthma and COPD, where enhanced SubP and/or tachykinin signaling is potentially pathogenic, a decrease in SubP-mediated secretion in CF might be of detriment [99]. However, restoring SubP-mediated signaling through tachykinin agonists seems like an ineffective strategy in CF because evidence suggests that the defect in SubP-mediated glandular secretion in CF is due to loss of CFTR [100]; thus, CFTR correctors and/or modulators would be required. Even if such a strategy was pursued, the pro-inflammatory effects [101] and potential bronchoconstricting effects [72–75] of tachykinins make this a less appealing option.

### Other Kinins

Recently a new gene and associated peptides have been added to the kinin group, although they do not entirely fulfill the requirements to be classical neuropeptides [69, 102]. These peptides have structural similarity with the known tachykinins but are present in a large variety of tissues and organs and are synthesized mainly by cells of the hematopoietic lineage [69, 102]. In humans, hemokinin 1 (HK-1), together with endokinins (EK) A, B, C and D, which originate from the different splice variants of the TAC4 gene (preprotachykinin C), have been found [102]. Due to the wide spread of HK-1 in the body, and its preferential affinity to the NK-1 tachykinin receptor, it has been implicated in many diseases (reviewed elsewhere, [103–105]) including asthma and possibly COPD [106–108]. HK-1, EKA and B pro-contractile effects have been shown in ex vivo bronchi both in humans and guinea pigs, although seemingly these effects are mediated through different receptors [109]. Very recently HK-1 has also been shown to cause degranulation of the human mast cell line leukocyte adhesion deficiency-2 (LAD2) [110]. These findings suggest that HK-1 might play an important role in the pathogenesis and symptomology of asthma and COPD.

### Vasoactive intestinal peptide (VIP)

VIP belongs to the glucagon/secretin gene family, with its own VIP gene that transcribes to two prepro-VIP precursors, giving rise to 3 peptides (in humans) – VIP, peptide histidine methionine 27 (PHM-27) and peptide histidine methionine 42 (PHV-42) [111]. Major functions of VIP include airway smooth muscle relaxation [112], stimulation of mucus secretion from airway glands and goblet cells [113], and vasodilation [112, 114]. The effects of VIP on mucus secretion in humans are complex as an inhibitory effect of VIP on cholinergic-mediated mucus secretion in human submucosal glands has been reported [115].

#### Asthma

Some evidence suggests a functional loss of VIP-innervation to the airways in asthma [116], although this may be secondary to inflammation. It was speculated that the loss of VIP-innervation to the airway diminished the amount of bronchodilation mediated by the nervous system. Athari et al. also found that mucus hypersecretion and muc5AC mRNA were significantly decreased when enzyme-degradation resistant VIP was delivered to the airways of mice with experimentally induced asthma [117]. Although speculative, this finding might suggest that VIP has an inhibitory role on muc5AC expression in asthma. However, perhaps inconsistent with this speculation is data from the pancreas suggesting that VIP increases muc5AC expression [118]. Although we were not able to find any studies concerning the effects of VIP on muc5B regulation and/or expression in asthma, since muc5B might be decreased in asthma [119], and VIP might also be decreased, then one speculation is that VIP directly stimulates production of muc5B.

In 2003, Linden and colleagues examined the effects of a VIP agonist in asthma and found a short, but effective, bronchodilatory effect [120]. Others have shown that VIP possesses potent anti-inflammatory effects and inhibits eosinophil migration [121]. These properties make it an appealing therapeutic candidate for asthma. However, due to short plasma half-lives, VIP analogs have been met with limited enthusiasm clinically [122]. Efforts to modify formulation and delivery have been ongoing. Very recently, one agonist with sustained release (PB1046) has undergone further development for pulmonary hypertension [123]. Perhaps a renewed interest in VIP analogs will reinvigorate efforts to examine their therapeutic potential in asthma.

#### COPD

Increased serum levels of VIP might be a marker of acute exacerbations in COPD [124], although whether they are a cause or consequence is unknown. An argument for consequence is derived from studies suggesting a beneficial effect of inhaled VIP on quality of life in COPD [125], as well as evidence indicating a protective role of VIP against pulmonary hypertension in COPD. However, an argument for causal relationship might be inferred from studies demonstrating that chronic bronchitis, which is a common feature of COPD [126], is associated with increased VIP innervation to the mucus glands [127]. The authors suggested that increased VIP innervation to the mucus glands was linked to increased sputum production.

No studies were available that described VIP-mediated activation of goblet cells or glands in either humans or animal models of COPD. Similarly, we were unable to identify any studies that examined the effects of VIP on muc5AC or muc5B expression in COPD airways. However, receptors for VIP (vasoactive intestinal peptide receptor 1 (VPAC), vasoactive intestinal peptide receptor 2 (VPAC2)) were elevated in the epithelium and glands in biopsies from smokers with chronic bronchitis [128]. This finding might suggest that regulation of mucus secretion by VIP is altered in COPD. Additionally, since CFTR dysfunction is involved in COPD pathogenesis [56], one speculation is that VIP-mediated submucosal gland secretion, which is dependent upon CFTR [129], is impaired in COPD.

Very few studies have examined the therapeutic potential of VIP in COPD. As highlighted above, PB1046 is a VIP agonist with sustained release that is being developed for pulmonary hypertension [123]. Because pulmonary hypertension is common in COPD [130], it is possible that PB1046 will be of clinical value in COPD. However, given that increased VIP innervation to the mucus glands in COPD is proposed to contribute to increased sputum production [126], VIP agonists might be of mixed benefit.

#### Cystic fibrosis

Studies suggest that VIP nerve distribution and density are decreased in the airway epithelium, submucosal glands, alveolar walls and blood vessels of people with CF [131]. The decrease in VIP distribution was proposed to be secondary to infection and inflammation. It has also been shown that CF airway glands do not respond to VIP stimulation [129]. This lack of response has been interpreted to indicate that VIP stimulates gland secretion through CFTR-dependent mechanisms [132]. The authors suggested that the lack of VIP-mediated gland secretion might promote a hyper-inflammatory airway environment that contributes to CF lung disease. Interestingly, in mice, lack of VIP evokes CFTR dysfunction, creating a CF-like disease [133]. Together, these finding suggests a potential reciprocal relationship between VIP signaling and CFTR function.

Since VIP is ineffective at stimulating gland secretion in CF, then it seems an unlikely candidate to mediate the increased muc5B concentrations (which is largely expressed in the glands and to a lesser extent in surface goblet cells [134]) that are observed during CF exacerbations [135]. It is also unlikely that VIP agonists would be therapeutically beneficial for restoring glandular secretion in CF, since VIP-mediated secretion is dependent upon CFTR [132]. Thus, co-administration with CF correctors or potentiators would be necessary. However, the proposed anti-inflammatory and bronchodilator properties of VIP [121] make it a therapeutic option worth exploring.

### Calcitonin gene-related peptide (CGRP)

CGRP is a member of the calcitonin gene family related neuropeptides and its precursors are encoded by the calcitonin II gene [136, 137]. CGRP-alpha and -beta precursors are cleaved into two 37aa-long isoforms: α−CGRP and β−CGRP respectively [21, 114, 138]. CGRP is predominantly expressed in the central and peripheral nervous systems [139, 140]. CGRP-positive nerve fibers that innervate the airways originate from the trigeminal, nodose-jugular and dorsal root ganglia [138]. CGRP is also expressed in pulmonary neuroendocrine cells throughout the airway tree and in the alveoli [37, 141]. CGRP can induce mucus secretion in the airways, from both glands and goblet cells [142, 143]. CGRP also amplifies the pro-contractile effects of capsaicin [144] and electrical field stimulation [145].

#### Asthma

CGRP has long been suspected for having important modulatory role in asthma, due to its airway constricting capacity [146, 147]. Indeed, reports suggest that CGRP is increased in the bronchoalveolar lavage fluid of asthmatics and might contribute to the late phase asthmatic reactions following provocation by allergen inhalation [148]. CGRP-positive nerve fibers are also increased in animal models given viral infections, which are risk factors for asthma [149]. Interestingly, Larson and colleagues demonstrated that CGRP is expressed ectopically in mucus cells of ovalbumin (OVA)-sensitized Brown-Norway rats [150]. They suggested that this accumulation of CGRP might represent an additional releasing mechanism involved in quick hypersensitivity responses and mucus secretion. A recent investigation by Sui and colleagues found that a combination of CGRP and gamma-aminobutyric acid (GABA) are responsible for goblet cell hyperplasia and muc5AC induction in a murine model of asthma [39]. Using the same model, they also found that elimination of pulmonary neuroendocrine cells, which express CGRP, decreased the expression of goblet cells and muc5B.

There are currently several small molecules, as well as a monoclonal antibodies [151], that inhibit CGRP signaling [152]. Studies exploring the therapeutic potential of inhibiting CGRP signaling have focused largely on the cardiovascular system [153–156], with an emphasis on migraines. Many of those studies demonstrated an acute beneficial effect, however, liver toxicity associate with frequent use of CGRP small molecule inhibitors has hampered progress. To the best of our knowledge, there are no clinical studies that have examined the potential of CGRP receptor antagonists in clinical populations of asthmatics. However, with several tools available, examining inhibition of CGRP in the context of asthma should be achievable, even if only on a small scale. Based upon studies in animals, it is expected that inhibition of CGRP would alleviate some of the mucus phenotypes in allergic asthma [39].

#### COPD

Increased concentrations of CGRP have been identified in the sputum of people with COPD [157], where they have been speculated to play a role in promoting airway inflammation. Similarly, Gu et al. found increased frequency of CGRP-positive cells in the airways of people with COPD but decreased epithelial expression of CGRP receptors [158]. Although there are no studies that have specifically examined the effects of CGRP on muc5AC or muc5B expression in COPD, finding altered CGRP receptor expression in COPD airway epithelial cells suggests a possible influence of CGRP on muc5AC and muc5B expression. Correlative data also demonstrates a relationship between COPD, notch signaling, muc5AC mRNA, and pulmonary neuroendocrine cells (which synthesize CGRP among many other neuropeptides) [159].

We carefully examined the literature but were unable to find any studies that assessed the therapeutic potential of CGRP antagonists/blockers in preclinical or clinical COPD studies. However, given that CGRP can stimulate smooth muscle contraction in human airways [160], and has been implicated in mucus secretion in normal airways [142, 143], it is predicted that inhibition of CGRP might be of benefit in COPD. Yet, studies indicating that CGRP agonists possess specific anti-inflammatory properties, as well as vasodilatory properties [138], might suggest that inhibiting CGRP could be of potential negative consequence in COPD. Thus, clinical and/or preclinical studies that address the potential benefits of blocking CGRP (or the potential negative consequences) in COPD are necessary.

#### Cystic fibrosis

Although CGRP has also been shown to increase submucosal gland secretions in non-CF airways, it is an ineffective secretagogue in CF airways [161]. Of note, increased CGRP content has been found in the airway submucosal glands of humans with CF, as well as in mice, ferrets, and pigs with CF [161]. Taken together, these findings suggest that elevated CGRP in CF airways might be a compensatory mechanism to counter the lack of CGRP-mediated submucosal gland secretion. The authors also demonstrated an important role for CGRP in maintaining airway progenitor cells, which they speculated might be important for lung injury and repair in CF airways. Since CGRP participates in goblet cell hyperplasia and muc5AC induction in other disease models [39], it is possible that it is also involved in CF exacerbations, in which increased muc5AC has been noted [135].

With that said, it is difficult to predict whether inhibiting CGRP would be of significant benefit in CF, as one might anticipate mixed results (e.g. anti-inflammatory and vasodilatory properties versus involvement in mucus secretion). However, in the specific context of mucus, inhibition of CGRP might be of benefit in CF. Given the numerous CF animal models [162–165], preclinical studies examining this possibility are feasible.

### Neuropeptide Y (NPY)

Neuropeptide Y (NPY) is a 36 aa peptide that belongs to the F- and Y-amide gene family [21, 166, 167]. NPY is synthesized in both the peripheral and central nervous systems [168]. NPY has been implicated in a wide variety of autoimmune and inflammatory diseases, including asthma and COPD [37, 167, 169–171]. It plays an important role in regulation of airway blood flow [37], smooth muscle contraction [172], immune cell/mast cell modulation [167, 169] and secretion output from submucosal glands [173, 174].

#### Asthma

The role of NPY in asthma remains unclear. For example, NPY has been associated with stress-induced exacerbation in asthma [175–177]. From those studies, it was suggested that NPY facilitated inflammation, as its concentrations correlated with the number of leukocytes and eosinophils in the bronchoalveolar lavage fluid. Similarly, NPY through activation of its Y1 receptor, was demonstrated to be critical for the development of allergic airway inflammation in mice [178]. Specifically, NPY and Y1 receptor knock-out mice showed decreased eosinophils in the bronchoalveolar lavage fluid, as well as decreased circulating immunoglobulin E (IgE) levels, compared to wild-type mice under allergic conditions [178]. Additional studies have found that loss of *forkhead box protein P1* (*Foxp)1* and *forkhead box protein P4 (Foxp4)* in mice can induce ectopic expression of NPY in airway epithelia, resulting in airway hyperresponsiveness [172]. Loss of *Foxp1* and *Foxp4* are also associated with ectopic expression of muc5AC in the airway [179]. Thus, although a direct regulation of muc5AC by NPY has not been established, the two parallel ectopic expressions, suggest an association. Consistent with that, elimination of pulmonary neuroendocrine cells in a murine model of asthma, decreased *Npy* gene expression, which was associated with decreased goblet cell hyperplasia [39]. However, in contrast to the aforementioned studies, Chanez and colleagues found decreased expression of NPY in the airway smooth muscle, but not in the epithelium, of people with asthma [180]. The authors proposed that NPY might be protective in the airway, and that loss of NPY might contribute to mucus hypersecretion. Similarly, Lacroix and Mosimann reported that pretreatment with NPY decreased nasal obstruction and mucus secretion in allergic rhinitis [181].

Although the NPY modulation has been explored in obesity, alcoholism, anxiety, depression, epilepsy and pain (reviewed in [34, 182, 183]), its potential benefit in asthma is largely unknown and unexplored. The numerous small molecules that have been developed to either potentiate or inhibit NPY receptor offer promise for investigating NPY signaling in asthma [182].

#### COPD

In COPD, it has been reported that NPY expression is decreased in the lung epithelium, glands and smooth muscle tissue [171], and the authors speculated that such changes might contribute to COPD pathogenesis. In mice, secondary tobacco smoke exposure, which is considered a risk factor for COPD [184], increases the density of the NPY nerve fibers in the tracheal smooth muscle [185]. The increase in NPY nerve fibers was associated with airway hyperresponsiveness, suggesting a potential pathological role for NPY. Others have shown that NPY stimulates secretion of interleukin 6 from airway fibroblasts [186]. An implication therefore was that NPY might contribute to diseases like COPD and asthma, in which elevated interleukin 6 has been found in the sputum [187]. Interestingly, reports suggest that the concentration of muc5AC is increased in the sputum of smokers [188]; thus, one speculation is that NPY expression is positively correlated with muc5AC. Unfortunately, no studies have directly examined the effects of NPY of submucosal gland secretion or goblet cell degranulation, in COPD.

To address the role of NPY in COPD, studies utilizing antagonists or agonists in animal models or humans with COPD are required. Additionally, although no studies have examined the effects of modulating NPY in COPD, the NPY antagonist BIBO 3304 has purported beneficial effects in pulmonary hypertension [189]. Therefore, it is possible that NPY modulation might be of benefit in some people with COPD, such as those that have pulmonary hypertension [130].

#### Cystic fibrosis

NPY was of interest in CF due to its close chromosomal location to CFTR [190]. However, a linkage between the loci of CFTR and NPY was excluded, and thus it was concluded that NPY likely played little role in CF pathogenesis. The only other study that has examined NPY in CF was conducted in the olfactory system, in which it was found that NPY was increased in the olfactory epithelium of CF mice [191]. The increased NPY was associated altered expression of specific microvilli proteins, and it was suggested that olfactory function might be affected. Thus, whether NPY is involved in CF pathogenesis, or modulates mucus secretion in CF, remains unknown. The numerous CF animal models that develop airway disease similar to humans [162–165], as well as the multiple small molecules available that inhibit or potentiate NPY signaling [182, 189] offer ample opportunities to investigate the potential role of NPY in CF.

### Bombesins

The bombesin-like peptide gene family is currently comprised of 5 peptides, originating from two genes – the gastrin-releasing peptide gene and the Neuromedin-B gene [21]. Bombesins are synthesized by both the nervous system [192] and by pulmonary neuroendocrine cells [193], as well as other tissues [194]. In mammals, two of the peptides have been cloned and characterized – the 27 aa gastrin-releasing peptide (GRP) and the 10 aa neuromedin B (NMB), which act through their specific G-coupled receptors. For NMB, it is the subtype-1 receptor, and for GRP-27, it is the subtype-2 receptor [195–197]. Similar to the above described groups of neuropeptides, in humans, bombesin-like peptides (BLPs) have roles in many different physiological functions and pathological conditions – from glucose homeostasis to malignancies [195, 196, 198]. In the lungs, bombesins promote fetal lung development and maturation, as well as epithelial cell differentiation [37, 196]. They also play important roles in modulating airway physiology, including bronchoconstriction [199] and mucus secretion [200, 201], which we explore in more detail below.

#### Asthma

Recently, work by Sun and colleagues showed that the number of bombesin positive cells was increased in the airways of asthmatics [39]. Similarly, there have been several studies suggesting that exposure to tobacco products, which are triggers for asthma [202], increase components of BLP signaling in the lungs [203, 204]. Interestingly, the increased expression of BLP signaling components persisted after smoking ceased, suggesting a long-term sensitivity to the proliferative effects of BLPs. Additionally, bronchopulmonary dysplasia, in which bombesins have been implicated [205], is considered a risk factor for asthma [206]. Evidence also suggests that bombesin receptor-activated protein regulates muc5AC hypersecretion through neutrophil elastase [207]; neutrophil elastase been implicated in goblet cell degranulation and mucus hypersecretion in a rodent model of asthma [208]. While we found no studies describing the effects of bombesins on submucosal gland activation and/or regulation of muc5B in asthma, its ability to modulate glycoprotein secretion from whole tracheas under normal physiological conditions [201] suggests a potential role for it in pathophysiological conditions. Finally, bombesin antagonists have not been explored in asthma; however, several small peptide antagonists have been developed [209]. For example, monoclonal antibodies targeted against GRP have shown promise for disrupting bombesin signaling in human airways. Thus, using these tools to disrupt bombesin signaling might shed new light onto the role that they play in asthma.

#### COPD

Bombesins have been implicated in COPD [193], with evidence suggesting that there is greater release of bombesins from COPD airways. Consistent with that, greater numbers of pulmonary neuroendocrine cells, which express bombesins, have been observed in the airways of people with COPD [158]. Potential consequences of increased bombesin signaling in COPD include inflammation [210], bronchoconstriction [211], and altered lung injury repair [212]. Additionally, bronchopulmonary dysplasia, in which bombesins have been implicated, may increase the risk for development of COPD later in life [213]. Although the role of bombesins in modulating mucus secretion or muc5AC/muc5B regulation in COPD is unknown, given that bombesins are increased in COPD [193] and muc5B is also increased [188], then it is possible that either a direct or indirect relationship between bombesin signaling and muc5B expression exists. Bombesin antagonists have not been explored in COPD, and therefore preclinical studies focused on inhibiting bombesins might be of particular interest.

#### Cystic fibrosis

Increased expression of bombesin in neuroendocrine cells of people with CF has been reported [214, 215]. While no mechanisms explaining those changes were suggested, additional studies have shown that neuroendocrine cells express CFTR [43]. An implication therefore is that CFTR function is directly linked to pulmonary neuroendocrine function. Thus, given the proposed importance of neuroendocrine cells in asthma and COPD, it is possible that an undiscovered yet important role of neuroendocrine cells, and by extension bombesins, in CF exists. This might be especially true given their proposed involvement in submucosal gland secretion [201], muc5AC expression [207], inflammation [210], bronchoconstriction [211], and lung injury repair [212]. Utilizing available bombesin antagonists to explore the role of bombesins in CF might open the door to new therapeutics.

### Granins

Granins are a large family of putative neuropeptides found in the secretory granules of the chromaffin adrenomedullary cells in adrenal glands [216]. They display structural features close to the classical neuropeptides. They are also synthesized by neurons and are co-stored and/or released together with other neuropeptides; however, currently they are not considered to be among the classical neuropeptides [21, 216].

There are 6 genes, 7 prepro-peptides and 14 active cleaved peptides in the granin family [21, 22]. The most characterized of these peptides are chromogranin A and its derivative – vasostatin; chromogranin B, secretogranin II, and secretoneurin. Although the functions of granins are still being elucidated, secretoneurin has been shown to induce mucus hypersecretion in human airway epithelial cell lines (16HBE and NCI-H292) and induce expression of muc5AC [36], suggesting a modulatory role in mucus secretion. Consistent with that, chromogranin A is more commonly found in epithelial mucinous tumors compared to nonmucinous tumors [217].

#### Asthma

Very few studies have examined granins in asthma. One study reported increased serum levels of chromogranin A in a mixed population of individuals, some of which had asthma [218]. The premise of that study was to identify biomarkers of pulmonary neuroendocrine tumors. Recently, Sui and colleagues reported increased chromogranin A in the airways of mice sensitized to ovalbumin [39]. Elshafie and colleagues also reported that increased wheezing in a patient with asthma was associated with increased serum levels of chromogranin A [219]. It has also been reported that secretogranin II (known as chromogranin C), is a potent chemoattractant for eosinophils [220], which play a critical role in asthma [221]. The proposed signaling molecule mediating eosinophil chemotaxis in response to secretogranin II was cyclic AMP [220].

While we were not able to find any studies examining the role of granins in mucus hypersecretion in asthma, secretoneurin induces mucus hypersecretion in non-asthmatic human airway cells [36]. Moreover, studies suggesting that secretoneurin is co-released with SubP and CGRP raise the possibility that secretoneurin is pro-inflammatory [222]. If true, then inhibiting secretoneurin might be particularly useful in asthma. Since the cell surface receptor secretoneurin and other granins are currently unknown (Fig. 2), future studies focused on identifying the receptor responsible for granin signaling are important [223].

#### COPD

Elevations in serum levels of chromogranin A have been reported in people with COPD, and interestingly, the degree of obstruction correlated with chromogranin A concentrations [224]. The authors concluded that elevated chromogranin A reflected ongoing inflammatory processes in the airway. Circulating secretoneurin concentrations were also measured in people with COPD; compared to those with acute heart failure, people with COPD had decreased levels of secretoneurin [225]. It was proposed that secretoneurin was a “protective mediator” in people with heart failure. To the best of our knowledge, no studies have examined the potential contribution of granins in mucus-related phenotypes in COPD. However, it is possible that granins contribute to airway obstruction in COPD [224] through modulation of inflammation [222] and/or mucus secretion [36]. Additional studies are required to explore these possibilities.

#### Cystic fibrosis

Data indicate that pulmonary neuroendocrine cells expressing chromogranin A also express CFTR [226]. In neuroendocrine cells with diminished CFTR, neurosecretory properties were altered [226]. In follow-up study, Pan and colleagues determined that the number of pulmonary neuroendocrine cells were decreased in a mouse model of CF, resulting in diminished oxygen sensing [67]. An implication from those studies was that loss of CFTR might impair the function of neuroendocrine cells that express chromogranin A, and possibly alter chromogranin A expression and/or signaling. While we were unable to find any additional studies that investigated granins in CF, their potential for modulating inflammation [222] and mucus secretion [36] suggest that these neuropeptides might be of interest in CF airway disease.

## Conclusion

Neuropeptides continue to be a source of insight and complexity when it comes to airway disease. Although significant progress has been made in understanding the contributions of some neuropeptides to asthma, COPD, and CF, their specific contributions to mucus obstruction and/or hypersecretion is, in many ways, unknown. Therefore, the field of neuropeptides and mucus secretion is fertile with questions. Below we highlight a few potential future directions that can be expanded upon to advance the field.

A greater emphasis needs to be placed on understanding the role that neuropeptides play in modulating mucus secretion in “normal” airways. More specifically, studies that delineate the effects of neuropeptides on submucosal gland secretion versus surface epithelial cell secretion need to be addressed. Techniques including (but not limited to) epithelial cells cultured at the air-liquid interface [227], as well as methods developed by Wine and colleagues [228] and/or Welsh and colleagues [229] are suitable for such studies.

Additionally, given that muc5AC and muc5B expression profiles are affected in asthma [98], COPD [188], and CF [135], it is of interest to understand whether neuropeptides preferentially regulate expulsion of muc5AC and/or muc5B. Similarly, studies focused on determining whether neuropeptides influence the expression of muc5AC and/or muc5B at the protein or transcriptional levels will provide new insight regarding the regulation of mucins in both health and disease.

We found it surprising that there is relatively limited information available regarding the role that neuropeptides assume in regulating mucus secretion in asthma, COPD, and CF. Based upon our review of the literature available, more progress has been made in asthma compared to COPD or CF, but much work still remains. A few simple studies could narrow this knowledge gap. For example, using animal models or human samples derived from people with asthma, COPD, or CF, one can determine whether mucus secretion to a given neuropeptide is blunted or exaggerated. Similarly, examining how acute and/or repeated applications of neuropeptides affect expression of mucins (e.g., muc5AC, muc5B) in asthma, COPD, and CF would reveal a potentially causative role for neuropeptides in airway mucus phenotypes.

Additional studies might include the use of knockout or pharmacological approaches to assess the contribution of specific neuropeptides and/or their receptors to regulation of mucus secretion and expression. Moreover, a thorough investigation of the expression (both spatially and temporally) of neuropeptides and their receptors in the airway in both health and disease would provide additional insight regarding potential causative and/or modulatory roles in asthma, COPD, and CF.

Although it is difficult to select which neuropeptides are the most important and/or interesting to pursue, our review provides a road map to help guide researchers and clinicians. Clearly, species of interest will influence which neuropeptides can be readily studied, as the tools, models, and neuropeptides available vary according to species. In addition, there is less information regarding the signaling mechanisms of bombesins and granins. Thus, for these peptides, progress may require further efforts.

Mucus is a critical defensive mechanism [230]. Enhanced mucus secretion/production and/or abnormal mucus properties are associated with several airway diseases, as highlighted in this review. In order to find more specific targets and additional treatment options for people with asthma, COPD, or CF, a greater understanding of the regulation of mucus secretion by neuropeptides is undeniably important. We anticipate that future research aimed at investigating neuropeptide-mediated regulation of mucus secretion and expression will provide new insight into airway pathogenesis, as well as uncover new targets for therapeutic discovery.

## Abbreviations

aa: Amino acid
BB_1_: Bombesin subtype-1 receptor
BB_2_: Bombesin subtype-2 receptor
BLPs: Bombesin-like peptides
cAMP: Cyclic adenosine monophosphate
CF: Cystic fibrosis
CFTR: Cystic fibrosis conductance regulator
CGRP: Calcitonin gene-related peptide
DAG: Diacylglycerol
EK: Endokinins
FEV_1_: Forced expiratory volume
Foxp1: Forkhead box protein P1
Foxp4: Forkhead box protein P4
GABA: Gamma-Aminobutyric acid
GRP: Gastrin-releasing peptide
HK-1: Hemokinin 1
IgE: Immunoglobulin E
IP3: Inositol 1,4,5-trisphosphate
LAD2: Leukocyte adhesion deficiency-2
muc5AC: Mucin5AC
muc5B: Mucin5B
NK1: Neurokinin 1
NK2: Neurokinin 2
NK3: Neurokinin 3
NKA: Neurokinin A
NKB: Neurokinin B
NMB: Neuromedin B
NPY: Neuropeptide Y
OVA: Ovalbumin
PHM-27: Peptide histidine methionine 27
PHV-42: Peptide histidine methionine 42
RAMP1: Receptor activity modifying protein 1
RAMP2: Receptor activity modifying protein 2
SubP: Substance P
TAC1: Preprotachykinin A
TAC3: Preprotachykinin B
TAC4: Preprotachykinin C
VIP: Vasoactive intestinal Peptide
VPAC1: Vasoactive intestinal peptide receptor 1
VPAC2: Vasoactive intestinal peptide receptor 2
